# World Leprosy Day 2026: reflections on leprosy surveillance in Europe

**DOI:** 10.2807/1560-7917.ES.2026.31.3.2600030

**Published:** 2026-01-22

**Authors:** Paul E M Fine

**Affiliations:** 1 London School of Hygiene & Tropical Medicine, London, United Kingdom

**Keywords:** *Mycobacterium leprae*, leprosy, surveillance, Europe

The advent of World Leprosy Day 2026 on 25 January provides an opportunity to consider the surveillance of this ancient disease, in Europe as in the rest of the world. Leprosy, caused by infection with *Mycobacterium leprae*, still persists in much of the world, including all of Africa, all of South-east Asia and most of Latin America (except Chile). Arguably among the most stigmatising diseases, as reflected in the theme of this year’s World Leprosy Day: "Leprosy is curable, the real challenge is stigma", it is formally reportable in most if not all countries, but its surveillance poses a variety of problems, and available data are not complete. This is a major issue given that leprosy has been a target for global elimination, defined in various ways, since 1991 [1].

According to the World Health Organization (WHO), 172,717 new leprosy cases were diagnosed and reported, globally, in 2024 [2]. This indicates a massive decline from 752,417 new cases reported in 2000, and a continued gradual decline over the past decade, from 210,758 cases diagnosed in 2015. The huge decline between 2000 and 2015 was attributable largely to reports from India, and was seen as controversial by some [3]. In Europe the trend has been very different, from 18 new cases reported in 2015 increasing to 79 new cases in 2024 (most recent data available). A look at the data reported to WHO, as shown in the Table, suggests several complexities to these trends. The increase may be in part attributable to increased reporting given that the number of European countries submitting any (including nil) report rose from 28 to 35 over the past decade. But the numbers by country vary appreciably year to year, which suggests that reporting procedures have not been consistent. This is most obvious in the low numbers reported in 2020 and 2021 reflecting the disruption of reporting during the first 2 years of the COVID-19 pandemic. Beyond these issues, it is important to appreciate that leprosy is a very rare disease now in Europe, and the vast majority - if not all - of the cases recently identified are not autochthonous i.e. not attributable to infection acquired in Europe [4]. The increase in Europe thus reflects trends in numbers of people moving to Europe from ‘highly’ endemic countries in Africa, Latin America and South-east Asia. This is reflected in recent reports from 2025 of a case in Ireland where the patient was an immigrant who had lived in the Caribbean and Brazil, a case in Croatia where the patient, the first identified in Croatia since 1993, was a worker from Nepal, and in Romania where a woman from an unspecified Asian country was affected, constituting the first case identified in Romania since 1981 [5-7].

**Table:** Numbers of new leprosy cases reported globally, and in Europe, to the World Health Organization, 2015–2024

Year	Total number of new cases reported globally	Europe			
		Number of countries reporting	Number of countries with new cases reported	Total number of new cases reported	Numbers of newly reported cases, by country (n)
2015	210,758	28	5	18	Germany (2), Israel (2), Portugal (2), Spain (8), UK (4)
2016	214,783	31	7	32	Belgium (3), Germany (2), Italy (12), the Netherlands (5), Portugal (4) UK (5), Uzbekistan (1)
2017	210,671	25	5	33	France (8), Italy (8), Kazakhstan (1) the Netherlands (3), Spain (9), UK (4)
2018	208,617	23	9	50	Belgium (1), France (9), Italy (5), the Netherlands (2), Portugal (5), Russia (3), Spain (7), Sweden (11), UK (7)
2019	202,185	21	7	42	Belgium (2), France (10), Malta (1), Portugal (6), Sweden (9), Tajikistan (12), UK (2)
2020	127,396	28	6	27	France (7), Ireland (1), Israel (2), the Netherlands (4), Russia (6), Sweden (7)
2021	140,594	16	2	14	France (12), Germany (2)
2022	174,087	36	11	53	Andorra (1), France (18), Germany (3), Italy (3), the Netherlands (1), Norway (1), Spain (10), Tajikistan (7), Turkey (2), Ukraine (5), UK (2)
2023	182,816	34	9	33	France (6), Germany (1), the Netherlands (2), Russia (1), Spain (7), Sweden (8), Turkey (2), Ukraine (3), UK (3)
2024	172,717	35	11	79	Denmark (1), France (19), Germany (1), Ireland (1), Portugal (17), Spain (8), Sweden (8), Turkey (6), Ukraine (3), UK (12), Uzbekistan (3)

Source: Data extracted from annual reports in Weekly Epidemiological Record.

UK: United Kingdom.

Identification of the location of transmission of *M. leprae* is itself difficult, given that we have no good test for infection, and the incubation period to clinical manifestations is long and variable, ranging from a few years to decades. Transmission is thought to occur primarily by respiratory secretions from multibacillary individuals, but this may occur during the incubation period, and the exact point in time is thus difficult to infer, even with careful history taking and critical analysis. Cases are sometimes classified as autochthonous or non-autochthonous on the basis of place of birth – but it is of course possible for someone born in Europe to travel to and become infected in some other ‘highly’ endemic part of the world and then return to their home in Europe during the incubation period. The United Kingdom (UK) provides a good example, as leprosy has been closely monitored there for many decades [8]. Despite hundreds of cases identified over the past 50 years, the last case attributable to local transmission was diagnosed in 1954, and concerned the 9-year-old daughter of an immigrant with leprosy who had contracted their infection in Brazil. The child had never been outside the UK so there was definite evidence of local transmission [9]. Recent studies of trends in Spain [10] and northern Portugal [11] illustrate the growing circumstantial evidence that infection transmission has stopped in those countries.

The rarity of leprosy in contemporary Europe means that cases may go undetected, and the diagnosis is often considerably delayed. Clinicians need to be reminded that the disease does still exist and should be included in differential diagnosis of skin lesions and nerve involvement. It also means that policies on case management and reporting are inconsistent between countries. There have been calls for international leprosy agencies to assist in the development and promulgation of consistent policies [4].

One of the many outstanding issues about leprosy is the question of why there has been no evidence of infection transmission in northern Europe over several decades, despite the presence of hundreds of apparently infectious (multibacillary) cases among immigrants, themselves attributable to infections contracted abroad. Whether this is attributable to absence of infection transmission, or to the failure of locally acquired infections to manifest as disease, is among the most important outstanding questions concerning this ancient and fascinating disease. It is hoped that development and application of sensitive and specific tools to identify infection by *M. leprae* may one day be able to address this problem, but tests to date have not proven able to identify early infection [12].

## Note

Paul E M Fine is Emeritus Professor of Communicable Disease Epidemiology at the London School of Hygiene & Tropical Medicine (LSHTM), United Kingdom. Over 26 years, from 1978 to 2006, he was Principal Investigator of a large study of leprosy in Malawi, known as the “Karonga Prevention Study”.
